# A review of factors influencing the stress response in Australian marsupials

**DOI:** 10.1093/conphys/cou027

**Published:** 2014-07-23

**Authors:** Stephanie Hing, Edward Narayan, R. C. Andrew Thompson, Stephanie Godfrey

**Affiliations:** 1 School of Veterinary and Life Sciences, Murdoch University, Murdoch, WA 6150, Australia; 2 Environmental Futures Research Institute, Griffith School of Environment, Griffith University, Southport, QLD 4222, Australia

**Keywords:** Australia, conservation, glucocorticoids, marsupials, stress

## Abstract

Many Australian marsupials are threatened species. In order to manage *in situ* and *ex situ* populations effectively, it is important to understand how marsupials respond to threats. Stress physiology (the study of the response of animals to challenging stimuli), a key approach in conservation physiology, can be used to characterize the physiological response of wildlife to threats. We reviewed the literature on the measurement of glucocorticoids (GCs), endocrine indicators of stress, in order to understand the stress response to conservation-relevant stressors in Australian marsupials and identified 29 studies. These studies employed a range of methods to measure GCs, with faecal glucocorticoid metabolite enzyme immunoassay being the most common method. The main stressors considered in studies of marsupials were capture and handling. To date, the benefits of stress physiology have yet to be harnessed fully in marsupial conservation. Despite a theoretical base dating back to the 1960s, GCs have only been used to understand how 21 of the 142 extant species of Australian marsupial respond to stressors. These studies include merely six of the 60 marsupial species of conservation concern (IUCN Near Threatened to Critically Endangered). Furthermore, the fitness consequences of stress for Australian marsupials are rarely examined. Individual and species differences in the physiological stress response also require further investigation, because significant species-specific variations in GC levels in response to stressors can shed light on why some individuals or species are more vulnerable to stress factors while others appear more resilient. This review summarizes trends, knowledge gaps and future research directions for stress physiology research in Australian marsupial conservation.

## Abstract (precis)

Conservation of Australian marsupials requires an understanding of how they respond to threats. Measuring stress hormones is an important approach to characterise animals' response to threats but this approach is underutilised in marsupials. This review summarises how an improved understanding of the stress response in marsupials can benefit conservation.

## Introduction

Characterization of the stress response of animals, the physiological reaction to challenging stimuli, is essential to conservation because these processes underpin how wildlife responds to environmental change (Reeder and Kramer, 2005; Killen *et al.*, 2013; Madliger and Love, 2014). For this reason, the field of wildlife stress physiology is increasingly being recognized as an integral component of conservation physiology (Cooke *et al.*, 2013; Kersey and Dehnhard, 2014) and a key approach to improve wildlife welfare and management *in situ* and *ex situ* (Monfort, 2003; Bradshaw, 2010; Cooke *et al.*, 2013). However, stress physiology remains an underused approach in Australian marsupial conservation.

An extensive body of literature highlights the complex roles of the stress endocrine system in the physiological responses of wildlife to stressors. Stress can be stimulatory (optimizing physiological systems for action), preparative (priming systems for action) or inhibitory (especially chronic stress, which can cause permanent dysfunction of the endocrine stress response and lead to suppression of functions such as reproduction; Narayan and Hero, 2014a, 2014b). The stress response is a complex and vital physiological mechanism that enables individuals to cope with stressful situations (Munck and Náray-Fejes-Tóth, 1994; Sapolsky *et al.*, 2000). The complexities and importance of stress physiology have been recognized in many mammalian taxa (see reviews: marine mammals, Fair and Becker, 2000; carnivores, Young *et al.*, 2004; felids, Brown, 2006; primates, Muehlenbein, 2009) as well as birds (Palme *et al.*, 2005), amphibians (Gabor *et al.*, 2013; Narayan, 2013) and fish (Iwama *et al.*, 2011; Baker *et al.*, 2013). However, in comparison to other taxa, relatively little is known about the response of Australian marsupials to conservation-relevant stressors and the conservation implications of the marsupial stress response.

Stress physiology in Australian marsupials is of particular interest owing to their conservation status, evolutionary importance and unique physiological characteristics. Over 40% of the 142 (60 of 142) extant Australian marsupial species are of conservation concern (Kennedy, 1992). Marsupials also occupy a unique evolutionary niche (Armati *et al.*, 2006). Thus, investigation of how marsupials respond to stressors can provide further insights into the evolution of the physiological stress response, an area of ongoing scrutiny in evolutionary ecology (Boonstra, 2013). Australian marsupials are the most diverse extant marsupial radiation (Johnson, 2006) and possess many unique physiological characteristics, such as adaptations to specific climatic envelopes (Bradshaw, 1983), which may facilitate distinct physiological stress responses. Stress physiology is also a neglected area of research in marsupials from the Americas; however, for the purposes of identifying trends relevant to Australian marsupial conservation, this review focuses on the Australian context.

Marsupials are exposed to a suite of potential stress factors (McEwen, 2005; Bradshaw, 2010), such as habitat loss and increased predation pressure (Claridge *et al.*, 2007). These factors have contributed to nationwide decline and extinction of native species (Johnson, 2006). In order to manage and conserve marsupials into the future, it is crucial to characterize their physiological response to stress factors (Narayan *et al.*, 2013). Understanding responses to extrinsic (environmental) stressors is particularly important, because these types of factors have been found largely to determine extinction risk among Australian marsupials (Fisher *et al.*, 2003). Insights into marsupial stress physiology may also improve outcomes for injured and orphaned marsupials, because stress has been implicated as a major challenge to their successful care and survival (Jackson, 2007).

The aim of this review is to identify how stress physiology can be applied to the conservation of Australian marsupials. The stress physiology literature was examined to find studies on the stress response of marsupials to conservation-relevant stressors and those which highlighted the consequences of stress in marsupials. We surveyed the literature in view of three key questions. (i) What stress parameters and methods have been used for glucocorticoid quantification in marsupials? (ii) How have these tools been applied to identify significant stressors relevant to marsupial conservation? (iii) What are the significant knowledge gaps that require future research? We sought to evaluate whether stress physiology can be used to identify risk factors, aid management and improve conservation outcomes for threatened species of Australian marsupials.

## The physiological stress response

The stress response involves the production of neuroendocrine mediators (adrenaline, noradrenaline and glucocorticoids) and is essential to maintain allostasis (homeostasis through change) during exposure to a stressor (McEwen, 2005; Wikelski and Cooke, 2006). The hypothalamic–pituitary–adrenal (HPA) axis, the neuroendocrine pathway underpinning the physiological stress response, is highly conserved across vertebrates, including eutherian mammals, marsupials and monotremes (McDonald, 1977; Nesse and Young, 2007). Several texts (Downs, 1980; Moberg and Mench, 2000) and reviews have outlined the HPA axis in detail (e.g. Reeder and Kramer, 2005; Sheriff *et al.*, 2011). In summary, the HPA axis operates as a negative feedback system. Secretion of corticotrophin-releasing hormone from the hypothalamus stimulates release of adrenocorticotrophic hormone (ACTH) from the pituitary gland. Adrenocorticotrophic hormone promotes the release of many neuroendocrine mediators (Moberg and Mench, 2000), including the focus of this review, glucocorticoids (GCs) from the adrenal cortex (Möstl and Palme, 2002). The GCs (mainly cortisol and/or corticosterone) have complex interactions with virtually all biological processes (McLaren *et al.*, 2007). As for eutherian mammals, GCs in marsupials are known to regulate a suite of biological processes from protein, carbohydrate and fat metabolism (Bradley and Stoddart, 1990) to essential renal (McDonald and Bradshaw, 1993), neurological (McAllan, 2006), cardiorespiratory and reproductive functions (Shaw *et al.*, 1996). Ultimately, GCs influence wildlife health, fitness and survival (Jessop *et al.*, 2013).

The HPA axis of marsupials shares many similarities with that of eutherian mammals (Hume *et al.*, 1989; Booth *et al.*, 1990). However, there are several unique characteristics which may constitute evolutionary adaptations to stressors that marsupials face in harsh habitats (McDonald, 1977). For example, in some macropod species, such as quokka (*Setonix brachyurus*), GCs appear to lack diabetogenic and nitrogen-mobilizing actions (Bradshaw, 1983; McDonald and Bradshaw, 1993). Early studies involving the removal of both adrenal glands (bilateral adrenalectomy) concluded that marsupials may be less dependent on the HPA axis for survival compared with eutherian mammals, because marsupials appeared to survive for longer periods post-adrenalectomy (McDonald, 1977). Early reports also mention that marsupials exhibit low levels of circulating GCs compared with eutherian mammals (Weiss *et al.*, 1979) and have low sensitivity to an exogenous ACTH stimulation test (McDonald, 1977; Weiss *et al.*, 1979). However, more recent studies have readily demonstrated a GC response to an ACTH stimulation test as part of the validation of non-invasive GC assays (Hogan *et al.*, 2012; Narayan *et al.*, 2013).

## Research on the physiological stress response in marsupials

### What stress parameters and methods have been used for glucocorticoid quantification in marsupials?

There are several methods for measuring stress in wildlife (Sheriff *et al.*, 2011). We have focused on GCs as physiological measures of stress because they provide a mechanistic understanding of the physiological stress response (Cooke *et al.*, 2013). The other major group of hormones released in the stress response are catecholamines (adrenaline and noradrenaline). However, it is difficult to measure catecholamines in wildlife because they are released almost instantaneously when a stressor is encountered and have a very short half-life of <30 s in the circulation (Medeiros and Wildman, 2013). Little is known about the excretion of catecholamines, and the known metabolites are unstable (Palme *et al.*, 2005). Therefore, catecholamines are infrequently applied to evaluate the stress response in wildlife (Stoddart and Bradley, 1991; Dehnhard, 2007). Behavioural and haematological parameters are also used as measures of stress in wildlife. However, they can vary widely among individuals and lead to inconsistent results (McKenzie *et al.*, 2004; Young and Deane, 2006). Particularly in the case of behavioural parameters, the underlying neuroendocrine stress response can occur in the absence of significant behavioural changes (Hogan *et al.*, 2011). In contrast, GCs mediate many of the consequences of stress for health, reproduction and survival (Cockrem, 2005; Reeder and Kramer, 2005). Glucocorticoids are also increasingly acknowledged as practical endocrine indicators of stress in many species and are currently being integrated into wildlife conservation worldwide (Möstl and Palme, 2002; Palme *et al.*, 2005; Sheriff *et al.*, 2011).

Endocrinology has undergone a revolution since Chester Jones *et al.* (1964) carried out the first study on GCs in Australian marsupials by collecting adrenal venous blood from brushtail possums (*Trichosurus vulpecula*). While invasive terminal sample collection was commonplace in the 1960s and 1970s and *post-mortem* sampling continues to be informative (e.g. Oates *et al.*, 2007), these methods are not always practical or ethical, particularly if studying cryptic and endangered species. In the last 20 years, minimally invasive sampling has become more widely practised (Kersey and Dehnhard, 2014). Innovative minimally invasive techniques to measure GCs and their metabolites have been broadly applied to identify stressors to wildlife (Sheriff *et al.*, 2011). Assays have been validated for use in various wildlife species to allow GCs to be quantified in blood, faeces, hair (Sheriff *et al.*, 2011), urine (e.g. Narayan, 2013) and even water (Gabor *et al.*, 2013).

**Figure 1:**
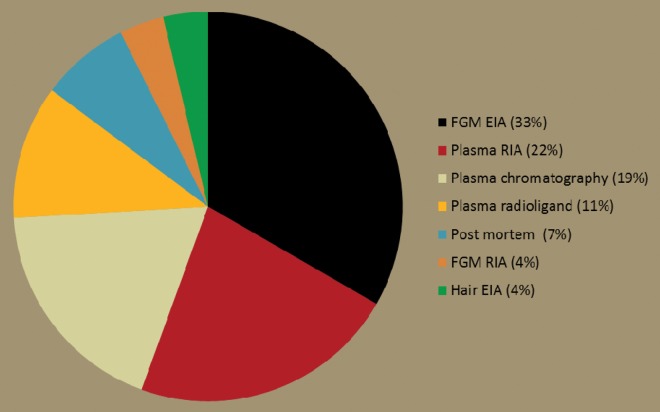
Stress parameters and methods used in marsupials. The most commonly measured stress parameter in marsupials are faecal glucocorticoid metabolites (FGM) using enzyme-immunoassays (EIAs; 33%). Radio-immunoassays (RIAs) and chromatography were also popular methods to measure plasma glucocorticoids.

Assays in marsupials initially relied on chromatographic techniques (Chester Jones *et al.*, 1964; Weiss and McDonald, 1966), but now radioimmunoassays (RIAs) and enzyme immunoassays (EIAs) are used, with EIAs having the advantage of lower resource costs, versatile equipment and lack of radioactive materials (Sheriff *et al.*, 2011). These assays have been employed in studies of Australian marsupials, including EIA (e.g. Narayan *et al.*, 2013) and RIA for faecal glucocorticoid metabolites (FGM; Oates *et al.*, 2007), EIA for plasma GC (e.g. Baker and Gemmell, 1999) and EIA for GC in hair (Brearley *et al.*, 2012). The FGM EIAs are the most common method used in studies of Australian marsupials (Fig. 1).

Key factors to consider when integrating stress physiology into marsupial conservation include considering what is to be measured, standardized protocols and validation (Möstl and Palme, 2002; Sheriff *et al.*, 2011). Firstly, if measuring GC in the circulation, it is important to consider that blood should be collected within 2–3 min of capture (Romero and Reed, 2005). This is rarely possible in marsupial field studies, because trapping and handling cryptic, sparsely distributed, often nocturnal marsupials with minimal resources in challenging terrain is not conducive to immediate collection. Hence, measuring FGM has proved to be a popular method. Recent research has explored the relative value of FGM analysis on fresh vs. older samples (by quantifying decay rates of FGMs; Evans *et al.*, 2013). Metabolites of GCs appear to be relatively stable in bilby faeces for up to 19 days (Evans *et al.*, 2013). However, as a precaution this validation needs to be done for each species owing to species specificity in decay rates of FGMs (Evans *et al.*, 2013).

It is critical that assays have been validated in the laboratory as well as physiologically (see reviews: Möstl and Palme, 2002; Millspaugh and Washburn, 2004; Touma and Palme, 2005; Sheriff *et al.*, 2011). For example, the ACTH stimulation test is a widely accepted method of biological validation because it demonstrates the cause-and-effect relationship between HPA axis activation and minimally invasive GC measurements. The ACTH stimulation test has been carried out to validate chromatography for plasma GC in Tasmanian devils (Weiss and Richards, 1971), quolls (Weiss and Richards, 1971), phascogales (Bradley, 1990) and quokkas (Bradshaw, 1983) and also to validate FGM EIAs in bandicoots (Dowle *et al.*, 2012), numbats (Hogan *et al.*, 2012), koalas (Narayan *et al.*, 2013b; Davies *et al.*, 2013a), bilbies (Narayan *et al.*, 2012) and wombats (Hogan *et al.*, 2011).

Interpretation of different parameters and assays should also be considered carefully. A range of potential methodological [e.g. sample collection, storage and extraction procedures, fraction of glucocorticoid measured, i.e. total, free, plasma corticosteroid-binding globulin (CBG) bound or albumin bound] and biological confounders (e.g. diurnal or seasonal fluctuations, reproduction, diet, pain and exercise) need also be considered when interpreting results and management implications (Millspaugh and Washburn, 2004; Palme, 2005; Lane, 2006; Evans *et al.*, 2013). The GC fraction measured is also noteworthy. For example, if the assay measures total cortisol, an increase in free cortisol may occur without an increase in total cortisol, depending on the rate of steroid metabolism (Hajduk *et al.*, 1992). Assays may measure non-protein-bound or free levels of the steroid. In circulation, GC can be bound to CBG, and CBG levels have been measured in marsupials to elucidate the amount of free vs. bound cortisol (Schmidt *et al.*, 2006). Free GCs are biologically active, but bound GCs may also exert their effect on cells via receptors for bound GCs (Sheriff *et al.*, 2011). The identity of the primary GC is important to consider because it too can differ between marsupial species (Oddie *et al.*, 1976).

As GC play a vital role in regulating metabolism, activity, reproduction and response to environmental challenges (Boonstra, 2013), fluctuations in GCs occur normally. Glucocorticoids can vary with diurnal rhythms (Than and McDonald, 1973; Allen and Bradshaw, 1980), reproductive status (Narayan *et al.*, 2013b) and season (Humphreys *et al.*, 1984; Bradley and Stoddart, 1992). For example, diurnal variations in GC have been documented in brushtail possums (Than and McDonald, 1973) and koala (Johnston *et al.*, 2013), and significant differences in FGM were noted between lactating and non-lactating female koalas (Narayan *et al.*, 2013). Seasonal variations in GCs have also been recorded in sugar gliders (Bradley and Stoddart, 1992) and scaly-tailed possums (*Wyulda squamicaudata*; Humphreys *et al.*, 1984). Such naturally occurring variation in GCs should be considered in order to avoid misinterpreting a normal GC fluctuation as a significant physiological response to a stressor. Study design and analyses must also acknowledge and try to account for these variables (Lane, 2006). Considering methodological and biological factors when designing minimally invasive GC methods will aid the incorporation of stress physiology into marsupial conservation.

### Identification of significant stressors to marsupials

**Figure 2:**
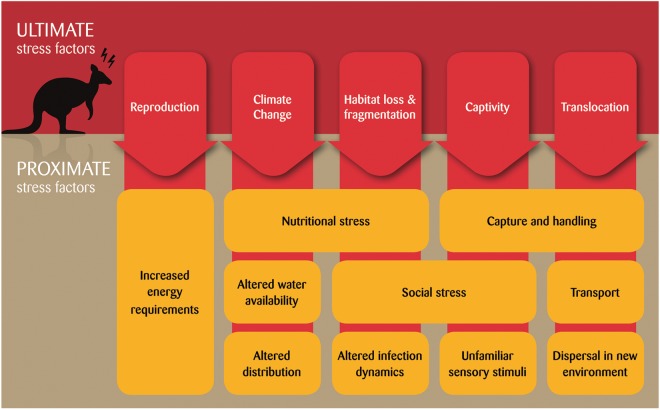
Ultimate and proximate stressors to marsupials.

A range of broad categories of stress factors have been identified in the marsupial stress physiology literature, including habitat loss and fragmentation, captivity, translocation and climate. It must be noted that each of these ultimate stress factors (root causes) may entail a number of potential proximate (immediate) factors. For example, habitat loss and fragmentation are associated with proximate factors including nutritional stress (Chapman *et al.*, 2007), altered social contact rates (Gillespie *et al.*, 2005) and changing patterns of parasite infection (Gillespie *et al.*, 2005; Fig. 2). Assessing the stress response of marsupials to these factors will allow mitigation of stressors which may have a detrimental effect on health, welfare and fitness and thereby increase conservation success.

#### Habitat loss and fragmentation

Habitat loss and fragmentation have a severe impact on wildlife population viability and the persistence of endangered species (McCallum and Dobson, 2002; Acevedo-Whitehouse and Duffus, 2009). Stress could be a consequence and could contribute to species decline in fragmented habitat (Brearley *et al.*, 2012; Johnstone *et al.*, 2012). Habitat loss and fragmentation may act as stressors through proximate mechanisms, such as reduced resource availability, increased competition, altered behaviour and social and disease stressors associated with changes in population density (Mbora and McPeek, 2009; Brearley *et al.*, 2013). Marsupials tend to experience greater physiological stress at higher densities compared with lower densities as measured by plasma GC (Thomas, 1990; Johnstone *et al.*, 2012). Northern quolls may be an exception, with no association found between plasma GC and the population density of quolls in the Kimberley region of Western Australia (Schmitt *et al.*, 1989).

Habitat fragmentation may also increase stress through the creation of more habitat edges. Squirrel gliders (*Petaurus norfolcensis*) living in edge habitats exhibited significantly higher cortisol compared with counterparts in interior habitats, as measured by GC metabolites in hair (Brearley *et al.*, 2012, 2013). Long-nosed bandicoots (*Perameles nasuta*), in contrast, had similar FGM concentrations in National Parks (continuous habitat) and suburban backyards (fragmented habitat; Dowle *et al.*, 2012), although other concurrent stressors pertaining to urban and wild locations may also explain these differences. Differences in physiological stress between marsupials in fragmented compared with continuous habitat may also underlie differences in immunocompetence and infection patterns (Johnstone *et al.*, 2012).

#### Captivity

Many species of marsupial are dependent on human care in zoos, wildlife parks and rehabilitation facilities (Kennedy, 1992). Numerous conservation programmes rely on captive breeding to supplement wild populations (Fa *et al.*, 2011). In order to maximize conservation outcomes and animal welfare, it is imperative to understand how marsupials respond to potential stressors in captivity. The captive environment may entail a range of potential biotic and abiotic stressors that they would not otherwise face in their native habitat, such as frequent human contact, abnormal social grouping, confinement, inability to exhibit natural behaviours, artificial light, alien odours and artificial substrates (Morgan and Tromborg, 2007). To an extent, captivity can also alleviate potential stressors, such as resource limitations, exposure to climatic extremes, disease and predators (Wielebnowski, 2003). Few studies have investigated the stress physiology of marsupials in relationship to captivity, but stressors identified to date include social isolation (McKenzie and Deane, 2005), social status (Stoddart and Bradley, 1991) and capture and handling (Narayan *et al.*, 2013). Health-related concerns, such as arthritis, dental disease, upper respiratory tract infections and associated veterinary procedures have also been found to be associated with increased mean FGM concentrations in captive bilbies (*Macrotis lagotis*; Narayan *et al.*, 2012).

Using FGM, Hogan *et al.* (2012) investigated the response of captive numbats (*Myrmecobius fasciatus*) to 20 key stressors classified in several categories: conspecific interactions (such as introduction or separation of animals), environment (such as rain and extreme temperatures), handling and health (including injuries, veterinary procedures and dietary changes) and anthropogenic disturbances (such as events and maintenance activities). Handling, conspecific interactions and health-related factors were found to elicit a significant physiological stress response, but interestingly, anthropogenic disturbances less so (Hogan *et al.*, 2012). The lack of a statistically significant response to anthropogenic disturbances does not necessarily indicate that these factors did not affect captive numbats (Hogan *et al.*, 2012). Indeed, with regular exposure, the animals may have habituated to anthropogenic disturbances (Bejder *et al.*, 2009).

Captivity can also alter or provide abnormal social groupings, leading to social stress. Stoddart and Bradley (1991) investigated social status and stress in captive male sugar gliders (*Petaurus breviceps*). The odour of a dominant male from another group elicited an increase in plasma GCs, whereas no changes in these stress parameters occurred with exposure to odour from a castrated male or a female (Stoddart and Bradley, 1991). These insights provide valuable information for the management of social groups in captivity.

While non-gregarious marsupials, such as numbats and sugar gliders, may experience stress associated with interaction with conspecifics, social marsupials may be stressed by social isolation. When tammar wallabies (*Macropus eugenii*), which are social marsupials that live in groups, were moved into captivity and isolated they exhibited a physiological stress response measured as an increase in FGM (McKenzie and Deane, 2005). As it was not possible to identify faecal samples individually from group-housed wallabies, comparisons between isolated and group-housed animals could not be made (McKenzie and Deane, 2005). Stress associated with social isolation is well characterized in other social mammals, including laboratory rodents (Weiss *et al.*, 2004), livestock (Carbajal and Orihuela, 2001) and primates (Sapolsky *et al.*, 1997). However, there appears to be a lack of studies on the impact of psychological stressors, including social stressors, on marsupials in captivity despite many marsupials (including many social macropod species) commonly being kept in zoos and wildlife parks.

Wildlife in captivity provide surrogate study specimens due to the logistical difficulties of working with free-ranging populations. Hence, a greater understanding of how Australian marsupials respond to captivity will aid the interpretation of results from experiments which use captive animals. Furthermore, measuring and monitoring stress in captive marsupials will improve conservation outcomes, particularly as the future of many endangered Australian marsupials relies on captive insurance populations.

#### Capture and handling

Capture and handling are relatively well-characterized stressors in aquaculture species (Baker *et al.*, 2013) and eutherian mammals, particularly laboratory animals and livestock (Grandin, 2007). A small number of studies have suggested that handling also elicits a stress response in both captive and wild-caught marsupials. Physiological indices, plasma GC and FGM indicate an acute stress response to handling in some species, including koalas (*Phascolarctos cinereus*; Hajduk *et al.*, 1992; Narayan *et al.*, 2013), numbats (Hogan *et al.*, 2012) and wombats (*Lasiorhinus latifrons*; Hogan *et al.*, 2011).

Behavioural reactions to capture and handling cannot always be accurate indicators of physiological stress. The behavioural reactions of captive wombats wane with regular handling, but increasing levels of FGM indicate that their physiological stress response does not diminish (Hogan *et al.*, 2011). This suggests learned helplessness, where the animal is in a state of stress but stops displaying associated behaviours because the stressor has continued (Hogan *et al.*, 2011). These results highlight the need for physiological measures of stress in addition to behavioural measures. Capture and handling are common in marsupial management, and the physiological stress response to these procedures has been shown to have consequences for health and survival in wildlife (Baker *et al.*, 2013). Faecal glucocorticoid metabolites can be used to investigate the impact of capture and handling stress in marsupial conservation and develop ways in which this stress could be managed.

#### Climate

Physiological stress associated with climatic factors such as extremes of temperature have been well documented in other mammalian species since the birth of the concept of stress (Selye, 1936). However, perhaps because it has been assumed that marsupials possess adaptations to harsh conditions (McDonald, 1977; Claridge *et al.*, 2007), there is little research into the adrenocortical stress response of marsupials to climatic extremes. Evaluating the response of marsupials to climate change using GCs will provide important information about how climate change may affect a wide range of biological functions. This approach will allow key stressors associated with climate change to be identified and predictions to be made about the impact of these stressors under different climate change scenarios.

King and Bradshaw (2010) characterized the physiological response of the critically endangered Barrow Island Euro (*Macropus robustus isabellinus*) to prolonged drought and associated factors, such as nutritional stress and lack of water, spanning 8 months during 1993–94. Euros exhibited increased plasma GCs and haematological changes, namely decreased eosinophils (Bradshaw, 2010; King and Bradshaw, 2010). These results suggested down-regulation or redistribution of the T-helper 2 arm of the immune system, which is responsible for defence against macroparasites (Ezenwa *et al.*, 2010; King and Bradshaw, 2010). The study suggested that immune function of the Euro might have been suppressed as a result of elevated corticosteroids. Thus, although marsupials possess many unique physiological adaptations, they are not immune to the impact of climatic stressors.

The lack of attention given to the adrenocortical response of marsupials to climatic factors is of concern, because stressors related to climate change are a significant threat to global biodiversity (Geyer *et al.*, 2011). Climate can affect marsupial health; for example, seasonal debility has been recorded in quokka in association with elevated plasma cortisol, weight loss, dehydration, parasite and bacterial infection (Miller and Bradshaw, 1979). Furthermore, climate change can influence the occurrence and distribution of wildlife populations (Loyola *et al.*, 2012; Haby *et al.*, 2013). Indeed, climate change has been suggested as a contributory factor in past, present and future range contractions and local extinctions of Australian marsupials, including northern bettongs (*Bettongia tropica*; Abell *et al.*, 2006; Bateman *et al.*, 2012), koalas (Adams-Hosking *et al.*, 2011) and quokkas (Gibson, 2001). Recent studies by Davies *et al.* (2013b, 2014) in koalas in south-west Queensland have found higher FGM in koalas at the arid edge of their range, particularly during drought and during winter. Their results suggested that koalas may be struggling to cope at the periphery of their range, and this may be exacerbated by variations in dietary composition with ongoing climate change (Davies *et al.*, 2013b, 2014). Investigations into the effects of climate change on wildlife, such as range contractions or (in some cases) expansions, are often approached from the standpoint of how climate change alters the ecology of the system (Thomas *et al.*, 2004), but associations between climate change and wildlife stress physiology remain an important area for further investigation (Wikelski and Cooke, 2006).

### What are the significant research gaps that require attention to strengthen Australian marsupial conservation?

**Figure 3:**
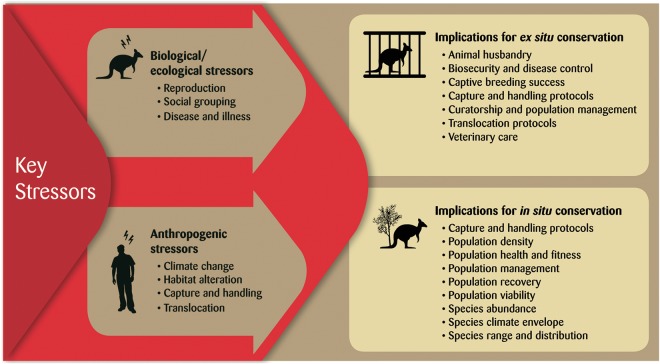
Key stressors to Australian marsupials documented in the literature and potential implications for *in situ* and *ex situ* conservation efforts.

Stress physiology is rarely applied to Australian marsupial conservation despite being long recognized as an important area of research in mammology and comparative endocrinology (McDonald, 1977), veterinary and human medicine (as models for disease and the evolution of physiological systems; Stein-Behrens and Sapolsky, 1992; Baudinette and Skinner, 2001). Major knowledge gaps remain, including a lack of studies focusing on marsupials of conservation concern and the need for investigations into the consequences of stress in marsupials (Fig. 3). In addition, reports spanning decades suggest that stress is also a major challenge to the rearing and rehabilitation of sick, injured and orphaned marsupials (Jackson, 2007). However, there appears to be a lack of published systematic investigations of stress physiology of marsupials in wildlife care. Young macropods are reported to be particularly vulnerable to mortality associated with dysfunction of the stress response (Hopwood and King, 1972). Hence, future insights into stress physiology in marsupials can facilitate improvements to *in situ* and *ex situ* management of marsupials.

In our view, based on the synthesis of current knowledge available on marsupial conservation, priority areas for stress physiology research in endangered marsupials include assay validation (particularly of minimally invasive methods, such as FGM) and quantifying the response of Australian marsupials to conservation-relevant stressors, such as introduced predators, invasive species, diseases, anthropogenic activities, habitat loss and fragmentation and climate change. We acknowledge the significant logistical challenges associated with studying the stress response in Australian marsupials, many of which are cryptic, nocturnal and threatened, including resource limitations (for example, time- and labour-intensive trapping and sample collection) and the only recent development of validated assays. Captive populations may provide feasible candidates for assay validation (Hogan *et al.*, 2011; Narayan *et al.*, 2012). Once key stressors can be identified, measures to manage stress can be trialled, and if successful, incorporated into management plans.

Physiological stress assessments in natural populations using conservation physiology tools are becoming immensely useful for understanding conservation challenges for other threatened wildlife in Australia (Kindermann *et al.*, 2012; Graham *et al.*, 2013). Marsupial conservation can benefit in a similar manner through the integration of stress physiology assessments into ecological monitoring programmes. Thus, networking and collaborations between field managers and stress physiologists will boost Australian marsupial conservation programmes.

#### Taxonomic coverage

Glucocorticoids have been used in 29 studies to understand the stress response to conservation-relevant stressors in 21 of the 142 extant species of Australian marsupials (15%; Table 1). The majority of species (60%) examined in these studies were listed as of least concern by the International Union for the Conservation of Nature (IUCN). There are an estimated 60 Australian marsupials of conservation concern (Kennedy, 1992), but glucocorticoids have been used to investigate the stress response of only one critically endangered (Stead-Richardson *et al.*, 2010) and two endangered species of Australian marsupial (Weiss and Richards, 1971; Hogan *et al.*, 2012; Table 1). Characterization of the physiological stress response of marsupials of conservation concern is needed because threatened species such as these are likely to be the most vulnerable to stressors; hence, these species may benefit most from the application of stress physiology to characterize the stress response and identify optimal management in the presence of threatening processes.

**Table 1:** Summary of studies using glucocorticoids to understand the stress response of marsupials

Species	IUCN	Captive/wild	Sample	Assay	Molecule	Keywords	Reference(s)
*Antechinus flavipes* Yellow-footed antechinus	LC	Wild-caught	Plasma	Radioligand assay	Total CORT	Semelparity	McDonald *et al.* (1981)
*Antechinus stuartii*^a^ Brown antechinus	LC	Wild-caught	Plasma	Chromatography	Total, free and CBG-bound CORT	Semelparity	Bradley *et al.* (1980)
*Antechinus swainsonii* Dusky antechinus	LC	Wild-caught	Plasma	Radioligand assay	Total CORT	Semelparity	McDonald *et al.* (1981, 1986)
*Dasyurus hallucatus* Northern quoll	EN	Wild	Plasma	Radioligand Gel electrophoresis	Total, free, CBG-bound and albumin-bound CORT	Species differences, reproduction, population density	Schmitt *et al.* (1989)
*Isoodon macrourus* Northern brown bandicoot	LC	Wild	Plasma	Equilibrium dialysis	Total, free and albumin-bound CORT and corticosterone	Season, species differences	Kemper *et al.* (1989)
*Lasiorhinus latifrons* Wombat	LC	Captive	Faeces	EIA	FGM (CORT)	Validation, handling	Hogan *et al.* (2011)
*Macropus robustus isabellinus* Barrow Island Euro	LC	Wild	Plasma Whole blood	RIA Haematology	Total CORT	Climate	King and Bradshaw (2010)
*Macrotis lagotis* Bilby	VU	Captive	Faeces	EIA	FGM (CORT)	Validation, acute and chronic stress	Narayan *et al.* (2012)
*Perameles nasuta* Long-nosed bandicoot	LC	Wild	Faeces	EIA	FGM (corticosterone)	Habitat, population density	Dowle *et al.* (2012)
*Phascogale tapoatafa* Brush-tailed phascogale	NT	Captive	Plasma	RIA	Total, free and CBG-bound CORT	Semelparity	Schmidt *et al.* (2006)
*Phascolarctos cinereus* Koala	LC	Captive and wild Wild-caught Wild	Faeces Plasma Whole blood Faeces	EIA RIA Haematology EIA	FGM (CORT) Total CORT FGM (CORT)	Validation, handling Capture Validation, climate	Narayan *et al.* (2013) Hajduk *et al.* (1992) Davies *et al.* (2013a, 2013b, 2014)
*Potorous gilbertii* Gilbert's potoroo	CR	Captive, Wild-caught	Faeces	EIA	FGM (CORT)	Reproduction, captivity	Stead-Richardson *et al.* (2010)
*Myrmecobius fasciatus* Numbat	EN	Captive	Faeces	EIA	FGM (CORT)	Validation, multiple stressors	Hogan *et al.* (2012)
*Phascogale calura* Red-tailed phascogale	NT	Wild-caught	Plasma	Chromatography Gel electrophoresis RIA	Total, free, CBG-bound and albumin-bound CORT and total corticosterone	Semelparity	Bradley (1990)
*Qyulda squamicaudata* Scaly tailed possum	DD	Wild	Plasma	Radioligand	Total, free and albumin-bound CORT	Season	Humphreys *et al.* (1984)
*Isoodon obesulus* Southern brown bandicoot	LC	Wild	Plasma	Radioligand Haematology	Total CORT	Population density	Thomas (1990)
*Petaurus norfolcensis* Squirrel gliders	LC	Wild	Hair	EIA	CORT	Habitat loss	Brearley *et al.* (2012)
Petaurus breviceps Sugar gliders	LC	Wild Wild	Plasma Whole blood via chronically placed catheter	RIA Equilibrium dialysis, gel electrophoresis	Total, free, CBG-bound and albumin-bound CORT Total, free, CBG-bound and albumin-bound CORT and catecholamines	Reproduction Social status	Bradley and Stoddart (1992) Stoddart and Bradley (1991)
*Macropus eugenii* Tammar wallaby	LC	Captive	Serum Serum Faeces	RIA EIA EIA	Total CORT Total CORT Total corticosterone FGM (corticosterone)	Season Validation, Social isolation, Feeding, Social isolation	McKenzie and Deane (2003) McKenzie *et al.* (2004) McKenzie and Deane (2005)
*Tarsipes rostratus* Honey possum	LC	Wild-caught	Faeces	RIA	FGM (CORT)	Captivity	Oates *et al.* (2007)
*Trichosurus vulpecula* Brushtail possum^b^	LC	Wild	Plasma	RIA	Total CORT	Translocation	Baker and Gemmell (1999)

Abbreviations: CBG, plasma corticosteroid-binding globulin; CORT, cortisol; EIA, enzyme immunoassay; FGM, faecal glucocorticoid metabolite; RIA, radioimmunoassay. International Union for the Conservation of Nature status: CR, critically endangered; DD, data deficient; EN, endangered; LC, least concern; NT, near threatened; VU, vulnerable. ^a^Selected papers, which reflect the complex links between stress and reproduction, have been included from the body of literature on semelparity in antechinus, because this has been reviewed previously (e.g. Bradley, 2003; Naylor *et al.*, 2008). ^b^Selected papers have been included from the literature on glucocorticoids in brushtail possums, because the foundations of this research dating back to the 1960s have previously been reviewed (e.g. McDonald, 1977).

#### Other neglected stressors

Many factors which have been found to elicit a significant stress response in other wildlife species have yet to be investigated in Australian marsupials. Challenges for marsupials, such as predators (Ramp *et al.*, 2005; Parsons and Blumstein, 2010) and invasive species (Dickman, 1996), have been investigated in terms of their behavioural and ecological effects, but the physiological response of marsupials to these threats has yet to be scrutinized fully. In these instances, stress physiology would allow more complete evaluation of the impact of these threats and thus assist in the design of more effective threat-management strategies. It is perhaps not surprising that these stressors remain unexplored given that experimental manipulation of these factors in marsupials is difficult and opportunities to do so are rare. An exception may be translocation stress, because translocations are widely used as a conservation strategy for Australian marsupials (Sheean *et al.*, 2012). Translocation entails a combination of many potential stressors, including capture and handling, transport and dispersal in a new environment (Teixeira *et al.*, 2007; Fig. 2), but few studies have explored the response of wildlife to these events during translocation (Franceschini, 2007) and how these can be designed to manage stress. Baker and Gemmell (1999) demonstrated that brushtail possums (*T. vulpecula*) experience stress following translocation, as indicated by elevated levels of plasma cortisol following capture and translocation. Given that marsupial translocations are an important and widely used conservation tool, a key question is: what aspects of translocation are most stressful, and consequently, how can translocation success be optimized? Most importantly, stress physiology tools can also be used to gauge the adaptation of animals post-translocation.

#### Understanding individual and species differences

The reasons underlying individual and species differences in the stress response are rarely examined in marsupials or wildlife in general (Morgan and Tromborg, 2007; Mason, 2010). Physiological differences between individuals and species are not random, in that differences can be determined by a myriad of factors, such as animal temperament, prior experience, environmental requirements and characteristics of biology (Wielebnowski, 2003; Bejder *et al.*, 2009; Mason, 2010). A key question is: what characteristics make some animals more resilient than others? The reasons for individual and species differences may indicate variation in vulnerability to decline and thus may shed light on desirable or undesirable traits and inform conservation priority setting and resource allocation. For example, plasma cortisol (free, CBG bound and albumin bound) in northern brown bandicoot (*Isoodon macrourus*) indicated that this species appeared to be less stressed by seasonal changes compared with sympatric marsupials, such as the northern quoll (*Dasyurus hallucatus*; Kemper *et al.*, 1989). *Isoodon macrourus* may be more resilient to climatic changes because they are generalists in terms of diet and habitat and do not experience the same breeding stress as other marsupials (Kemper *et al.*, 1989). Hence, in the case of extremes of weather, if resources for conservation mangement were limited, they could be prioritized for more specialized species, which may require more support, or in a triage situation, prioritized for generalists who are more likely to survive. Marsupial species also differ in their response to factors associated with reproduction, such as higher energy expenditure and intraspecific aggression. Antechinus species undergo semelparity while others, such as northern quoll (*D. hallucatus*), avoid fatal stress-related pathology associated with breeding (Schmitt *et al.*, 1989). The appropriate design of population-monitoring programmes may depend heavily upon this knowledge of species-specific stressors.

Individual differences in the stress response of Australian marsupial species have been explored by Narayan *et al.* (2012). Individual bilbies reacted differently to short-term stressors depending on the degree to which they had habituated to long-term stressors and their health condition (Narayan *et al.*, 2012). In contrast, Hogan *et al.* (2012) showed no significant variation among individual responses of captive numbats to the same stressors. An individual animal's stress response to characteristics of a captive environment may be influenced by a variety of factors, for example, past experience, time in captivity and nature of the contact (Morgan and Tromborg, 2007; Narayan *et al.*, 2013a). Potentially, with the advancement of molecular technologies, genetic markers associated with the variation in glucocorticoid data could be identified in marsupials to breed more resilient populations selectively. Human research has revealed that the stress response is moderately to highly heritable, and the neuropeptide Y gene has been identified to regulate individual variation in the effects of stress (Zhou *et al.*, 2008).

#### The consequences of stress

Marsupial stress physiology studies have largely concentrated on the essential first steps, such as biological and laboratory validation of assays and comparing individuals and populations of marsupials (Table 1). As small fluctuations in GCs are essential for survival, demonstrating changes in GC in itself does not necessarily indicate a potentially detrimental stress response. However, it is clear that prolonged high plasma cortisol (chronic stress) can reduce survival of marsupials (Bradley *et al.*, 1975). Incorporating GC monitoring into routine fauna monitoring of wild populations may provide insights into fluctuations between GC levels and population growth and fitness.

Acute vs. chronic stress can have different effects on fitness (Sapolsky *et al.*, 2000); hence, it is important to understand the consequences of both acute stressors, such as capture and handling, and chronic stressors, such as confinement (Stead-Richardson *et al.*, 2010). Indeed, there may be an interaction between responses to acute and chronic stressors, as observed in bilbies where individuals experiencing long-term stress associated with injuries and health issues demonstrated a greater FGM response to acute stressors (Johnstone *et al.*, 2012; Narayan *et al.*, 2012).

In order to determine whether a stressor poses a significant threat to an animal, further investigations are needed into associations between GC and biological functions (Madliger and Love, 2014), including immune defences, disease dynamics, reproduction and fitness (Moberg and Mench, 2000). Measuring these parameters in parallel to GC and comparing baseline GC with changes in response to specific stressors will help discern between normal vs. potentially detrimental variations in GC. It is crucial that links between GC, biological function and fitness are fully characterized to understand the impact of stressors on Australian marsupials, particularly species of conservation concern. For example, the health consequences of stress are many and varied (Sapolsky *et al.*, 2000), with serious implications for a variety of infectious diseases (Kriek, 1994; Narayan, 2013). In marsupials, stress has been associated with changes in body condition, bacterial and parasitic infection (Kemper *et al.*, 1989).

Stress may play a role in driving disease dynamics in Australian marsupials. For example, stress is implicated as a potential trigger for clinical disease in koalas infected by *Chlamydia* (Canfield *et al.*, 1991). Stress has also been suggested as a factor exacerbating *Toxoplasma gondii* infection, potentially contributing to the decline of Australian native fauna (Thompson *et al.*, 2010). In addition, *Trypanosoma* species of haemoparasites, which may play a role in the decline of the critically endangered woylie (*Bettongia penicillata*; Thompson *et al.*, 2010; Botero *et al.*, 2013), may be exacerbated by stress (Brown *et al.*, 2003). A small number of studies have been conducted in marsupials to investigate associations between stress and disease (Dowle *et al.*, 2012; Robert and Schwanz, 2013). However, we are yet to investigate and characterize fully the health and conservation implications of physiological stress in marsupials. By doing so, disease risk factors may be identified and mitigated to protect healthy populations for the future.

#### Reproduction

The relationship between stress and reproduction is relevant to marsupial conservation because chronic stress can limit breeding (Bradley *et al.*, 1980; Oates *et al.*, 2007); hence, it is important to consider associations between stress and reproduction for the sustainability of free-ranging populations and captive-breeding programmes. Chronic stress associated with breeding can be associated with fatal pathology (Dickman and Braithwaite, 1992; Bradley, 2003). For example, in *Antechinus* species and the red-tailed phascogale (*Phascogale calura*), the negative feedback system that regulates GC release is abolished during the breeding season, resulting in dysregulated and increased GC release and mass mortality (Bradley *et al.,* 1980; McDonald *et al.*, 1986; Bradley, 1997). A decrease in plasma CBG results in an increase in total free (biologically active) cortisol to levels which appear to be immunosuppressive, decreasing serum immunoglobulins (Bradley *et al.*, 1980). Consequently, gross *post-mortem* and histopathology reveal severe haemorrhagic gastrointestinal tract pathology, including ulceration (Bradley and Monamy, 1991), increased invasiveness of micro-organisms and parasites (Bradley *et al.*, 1980; Dickman and Braithwaite, 1992), liver pathology associated with *Listeria monocytogenes* (Barker *et al.*, 1978) and recrudescence of haemoparasites, such as *Babesia* spp. (Cheal *et al.*, 1976; Barker *et al.*, 1978).

The association between chronic stress and animal reproduction has also been determined experimentally. To determine the pattern of adrenal activity that was responsible for semelparity, Bradley *et al.* (1975) administered 10 mg/kg/day exogenous corticosterone to wild-caught male antechinus over 2 weeks to simulate chronic stress during the breeding season. The mortality rate of the treatment group was significantly higher than that of the control group (Bradley *et al.*, 1975). These processes may be affected by other environmental factors, since captive and wild brush-tailed phascogales (*Phascogale tapoatafa*) differ in their physiological stress response to reproduction; captive males escape the semelparous fate of their wild counterparts (Schmidt *et al.*, 2006). These insights indicate that conservation success for semelparous marsupial species may rely on mitigation of proximate stressors associated with reproduction, such as energy deficits.

Other dasyurid species, such as northern quoll (*D. hallucatus*), do not experience fatal stress-related pathology associated with breeding (Schmitt *et al.*, 1989). However, even in marsupial species that do not undergo semelparity, stress may influence breeding success, and stress physiology can be a highly informative tool for troubleshooting reproductive failure. For example, honey possums (*Tarsipes rostratus*) are vulnerable to chronic stress in captivity, and stress appears to be an important factor impeding reproductive success (Oates *et al.*, 2007). Few Gilbert's potoroo (*Potorous gilbertii*), Australia's most endangered marsupial, have been successfully bred in captivity, and Stead-Richardson *et al.* (2010) investigated whether stress may diminish reproduction in captive *P. gilbertii*. If this were the case, Stead-Richardson *et al.* (2010) surmised that captive potoroos would demonstrate significantly higher FGM compared with their wild counterparts, but their results suggested the reverse.

There are several possible explanations for differences in the relationship between stress and reproduction in different studies (captive and wild) and species, including the following: differences in protocols, methods and techniques; species differences in GC responses; length of time in captivity; characteristics of the captive environment; and the nature, frequency, duration and severity of stressors (Wielebnowski, 2003). Interpreting stress physiology and reproduction can also be problematic given the complexity of the interactions between GCs and reproductive hormones (oestrogens, progesterone and testosterone; Lane, 2006). Nevertheless, there is an extensive body of literature on stress and reproduction in other species indicating that stress can have a significant impact on reproductive success depending on a variety of individual, species, stressor and environmental factors (Tilbrook *et al.*, 2000; Karsch and Breen, 2009). For example, it has been consistently demonstrated that chronic stress reduces the secretion of gonadotrophins and inhibits reproduction in non-rodent mammals (Tilbrook *et al.*, 2000); hence, it is likely to be beneficial to marsupial conservation to consider stress physiology when evaluating the reproductive performance marsupials *in situ* and *ex situ*.

## Conclusions

Studies on the stress physiology of Australian marsupials have direct significance for the management of these unique species because they highlight significant factors that may influence conservation outcomes, i.e. reproduction, habitat loss, captivity, climate, capture, handling and translocation. There remain many other potential stressors that are yet to be investigated.

There is a broad range of situations where knowledge about the stress response could inform marsupial management plans. In captive populations, understanding what social groupings, housing conditions, environmental provisions and husbandry techniques incur more or less stress would help to improve welfare, health and fitness. For example, Hogan *et al.* (2011) demonstrated learned helplessness in captive wombats in response to handling and suggested that ‘gentling’, originally an equine training technique involving gradual, patient husbandry, may improve wombat management. Demonstration of the stress response of social marsupials to isolation (McKenzie and Deane, 2005) suggests that isolation should be avoided if possible, for instance if an individual requires off-exhibit treatment, the patient should be accompanied by conspecifics. The design of conservation protocols would also benefit from an understanding of the stress response; for example, insights into which stages of translocation are most stressful for marsupials and how provisions such as lighting, substrate and sound barriers might be used during transport to decrease the stress inherent in translocation. Stress physiology would also aid in evaluation of the efficacy of such measures.

In wild populations, understanding the stress response to major threats, such as habitat loss and climate change, is critical for conservation planning. Stress physiology research has indicated that marsupials such as koalas (Davies *et al.*, 2014) and squirrel gliders (Brearley *et al.*, 2012, 2013) are vulnerable at habitat edges, so resource allocation may prioritize these populations. Data can also be applied to the design of Australia's protected area network to strengthen the case for increased connectivity (Soulé *et al.*, 2004). The response of marsupials to stress factors may have a myriad of implications for *in situ* and *ex situ* conservation, but further studies are required to establish whether stress has a significant impact on the health and fitness of Australian marsupials, or indeed, if it contributes significantly to extinction risk and species survival.

While there is a body of literature on marsupial stress physiology spanning over 50 years, Australian conservation research and management programmes are yet to realize the potential benefits of stress physiology fully. Significant knowledge gaps remain in identifying how marsupials respond to stressors, the factors which modulate individual and population stress responses and the conservation implications of stress in marsupials. However, the rapid recent development of several non-invasive assays to measure stress parameters holds much promise for furthering this field of research into Australian marsupial conservation. Given urgent concerns about the future of endangered Australian marsupials, understanding how they respond to the stressors is critical for predicting how populations will cope and how best to manage them into the future.

## Funding

This work was supported by the Australian Academy of Science Margaret Middleton Foundation, Foundation of National Parks and Wildlife and the Holsworth Wildlife Research Endowment. S.G. was supported by an Australian Research Council DECRA (DE120101470). S.H. was supported by an Australian Postgraduate Award with a Murdoch University Strategic Top-Up Scholarship.
